# Vaccines for African swine fever: an update

**DOI:** 10.3389/fmicb.2023.1139494

**Published:** 2023-04-27

**Authors:** Hongliang Zhang, Saisai Zhao, Haojie Zhang, Zhihua Qin, Hu Shan, Xiulei Cai

**Affiliations:** ^1^Shandong Collaborative Innovation Center for Development of Veterinary Pharmaceuticals, College of Veterinary Medicine, Qingdao Agricultural University, Qingdao, China; ^2^College of Animal Science and Technology, Shandong Agricultural University, Tai’an, China

**Keywords:** African swine fever virus, epidemic and spread, vaccine, progress, review

## Abstract

African swine fever (ASF) is a fatal infectious disease of swine caused by the African swine fever virus (ASFV). Currently, the disease is listed as a legally notifiable disease that must be reported to the World Organization for Animal Health (WOAH). The economic losses to the global pig industry have been insurmountable since the outbreak of ASF. Control and eradication of ASF are very critical during the current pandemic. Vaccination is the optimal strategy to prevent and control the ASF epidemic, but since inactivated ASFV vaccines have poor immune protection and there aren’t enough cell lines for efficient *in vitro* ASFV replication, an ASF vaccine with high immunoprotective potential still remains to be explored. Knowledge of the course of disease evolution, the way of virus transmission, and the breakthrough point of vaccine design will facilitate the development of an ASF vaccine. In this review, the paper aims to highlight the recent advances and breakthroughs in the epidemic and transmission of ASF, virus mutation, and the development of vaccines in recent years, focusing on future directions and trends.

## 1. Introduction

African swine fever (ASF) is a highly contagious swine disease caused by the African swine fever virus (ASFV), in which the only natural host is swine (Wang et al., 2019). It is also the only large double-stranded DNA virus that infects domestic swine, wild boar, and blunt-edge ticks (Dixon et al., 2019). The virus has an icosahedral symmetry with a diameter of 200 nm and a concentric circle (Rowlands et al., 2009). Further, the ASFV genome contains a variable number of open reading frames (ORFs), ranging from 160 to 175, with approximately 125 conserved ORFs encoding more than 50 functional proteins (Sánchez-Vizcaíno et al., 2015a; Simões et al., 2019). The clinical symptoms of ASF are classified as acute, subacute, and chronic. Acute ASF has a rapid onset and short duration and is characterized by high fever, loss of appetite, cyanosis, severe internal bleeding, and a nearly 100% mortality rate (Pietschmann et al., 2015; Gaudreault et al., 2020), while less virulent strains cause milder clinical symptoms. ASF originated in Africa in the 1920s, and the disease spread to the Caucasus region of Georgia in 2007. From there, ASFV gradually spread to neighboring countries (i.e., Armenia, Azerbaijan, Russia, and Belarus), affecting both domestic and wild swine (Rowlands et al., 2008; Costard et al., 2009). Since August 2018, it has been detected in China as a genotype II highly virulent strain (Ge et al., 2018; Wen et al., 2019; Zhao et al., 2019). More than 40 outbreaks have occurred successively in the northeastern regions of Liaoning, Inner Mongolia, and Heilongjiang, causing unprecedented losses to the pig farming industry in China and seriously affecting national production and diet structure. Significant steps have been taken in recent years to curb the virus spread with the economy and international trade continued growth. Knowing the worldwide situation, prevalence, and transmission routes of ASFV is crucial for preventing the outbreak of diseases.

Genotype I was also known as the ESAC-WA genotype after 1957 when it was colonized in Europe, South America, and the Caribbean. By 2007, all countries (except Italy and Africa) had declared the eradication of ASF. Since the outbreak of ASFV genotype II in 2007, the world has been against ASF again, including Russia (2007), Ukraine (2012), Poland (2014), Belgium (2018), China (2018), Vietnam (2019), India (2020), and many other countries. Besides the wide range of transmissions, transmission routes are also very diverse. ASF is a viral disease transmitted via contact with infected swine and feces, either direct or indirect contact. Biological vectors such as ticks also accelerate the virus’s spread. Mechanical agents such as vehicles, tools, and human activities, as well as pig processing products, are also causative factors. Biological control is currently the most direct and efficient method of preventing ASF, which is superior to a vaccine.

The inactivated vaccines that are ineffective against ASF have been proven. Surprisingly, one of the attenuated live vaccines, ASFV-G-ΔI177L in Vietnam, is the first commercial ASF vaccine in the world (Tran et al., 2022a). However, live attenuated vaccine development is characterized by high costs, long cycles, and instability, which limit their rapid development. Researchers are currently focusing on genetically engineered vaccines such as subunit vaccines, vector vaccines, and DNA vaccines, while most experiments were limited to immunogenicity and did not carry out challenge experiments. Thus, developing a safe and effective ASFV vaccine is one of the top priorities against the virus. This review of the ASFV vaccine highlights the recent advances’ strengths and weaknesses and provides new research directions for promising progress.

## 2. Global scenario and prevalence of ASF

**Figure 1:**
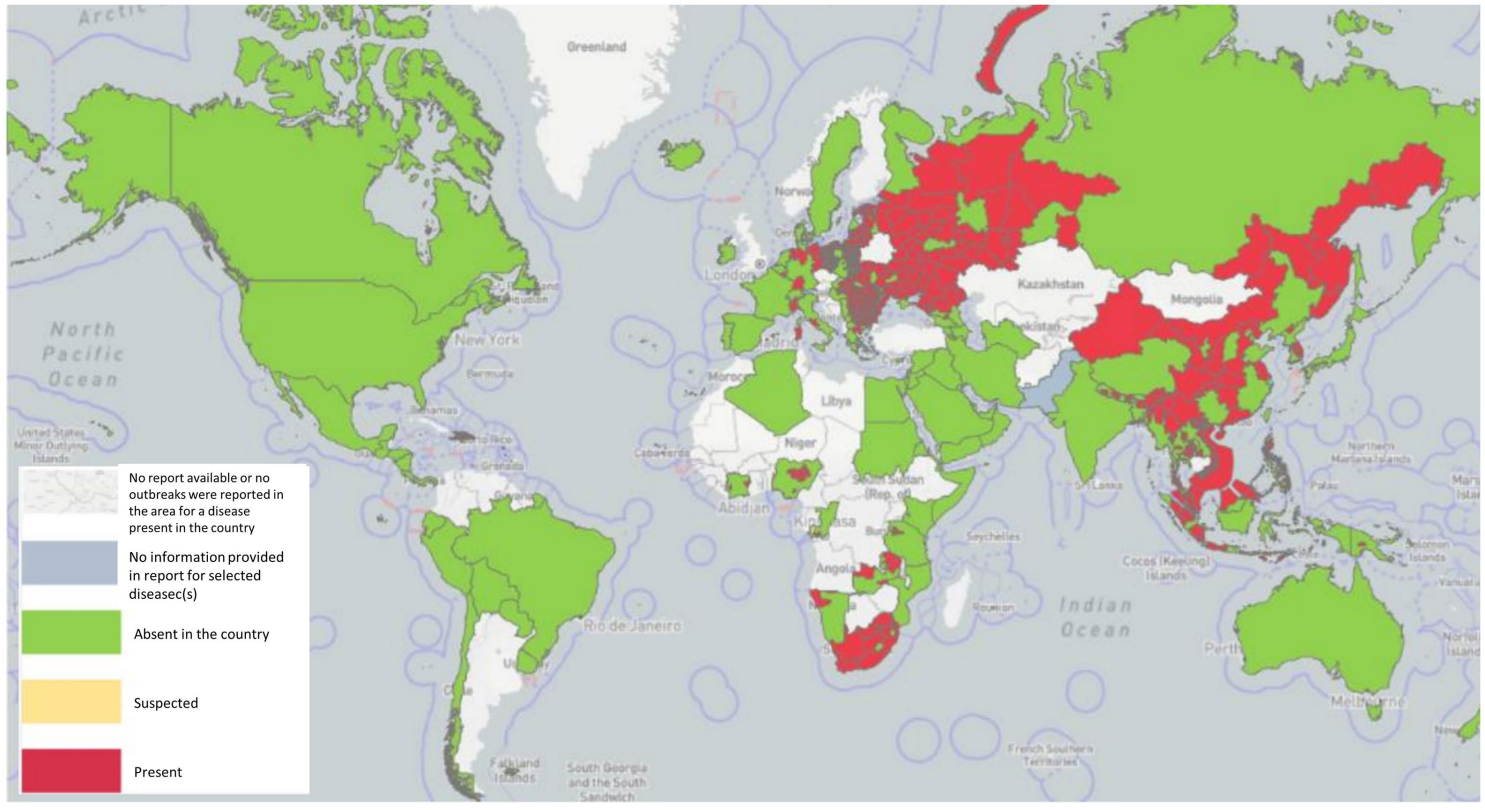
ASF has been reported in 45 countries globally since 2020 as of 29 September 2022 (Source: World Organization for Animal Health, World Health Organization, October 2022 line visit).

ASF is native to Africa, which maintains the virus by an ancient sylvatic cycle involving the natural hosts and vectors of the disease as well as domestic cycles with or without the involvement of natural vectors (Mulumba-Mfumu et al., 2019), has been recently reported in 32 countries since 2005. In Sardinia, there is genotype I, which is still endemic (Franzoni et al., 2020), whereas genotype II outbreaks occurred in Georgia in 2007, and from there, ASFV genotype II spread to neighboring countries, including Armenia, Azerbaijan, Russia, and Belarus, where it infected domestic swine and wild boar with a highly virulent strain of ASFV (Karger et al., 2019). A total of 9 countries in the continental European Union, such as Estonia, Lithuania, Latvia, Poland, the Czech Republic, Bulgaria, Belgium, Romania, and Hungary, have been severely affected by ASF from 2014 to 2018, and it persistently maintains spreading despite the efforts to control it. It caused high case-fatality rates in wild boar typical of the acute and subacute forms of the infection, particularly in newly infected areas (Martínez-Avilés et al., 2020). In just 2 years, from 2020 to 2022, a total of 16 EU countries reported the disease. Significantly, ASF was successfully eradicated from Belgium (March 2020) and the Czech Republic (April 2018; Baños et al., 2022). In August 2018, ASF was first proven in China (Liu Y. et al., 2021), and it quickly continued to spread to 31 provinces within a few months, resulting in a total of 178 ASF outbreaks. In 2019/2020, the disease spread to Oceania, with ASF reported in Timor-Leste, resulting in high mortalities in affected animals of small-scale pig farming (Berends et al., 2021; Phillips et al., 2021) and Papua New Guinea (Mighell and Ward, 2021). In early 2020, the first occurrence of ASF with high mortality in domestic swine in India (Arunachal Pradesh and Assam) was described, which is similar with the post-2007-p72-genotype II viruses reported from Asia and Europe, indicating the transboundary infection tendency of ASF outbreaks in the region (Rajukumar et al., 2021). In July 2021, ASF reappeared in the Americas after nearly 40 years of absence, first in the Dominican Republic and then in Haiti, with threats to animal health, livestock markets, and producer livelihoods (Jean-Pierre et al., 2022). Similarly, in January 2022, the Italian continent also notified the occurrence of ASFV genotype II after an absence of about 40 years (Beato et al., 2022; Iscaro et al., 2022). Meanwhile, two new countries have been affected: one of two in North Macedonia, caused by farms that were predominantly small-scale with high rates of turnover and the highest frequency of wild boar sightings (O’Hara et al., 2021), and the other of two in Thailand, caused by infected live and dead pigs, pork products, and wild boar semen, contaminated feed, and fomites (Thanapongtharm et al., 2022). The first reported ASF occurrence in Nepal was in March 2022, although the government had already banned the import of pigs and pork products from countries infected with ASFV on January 28, 2019 (Acharya and Wilson, 2020; Figure 1).

As shown in Table 1, since January 2020, ASF has been reported in five different regions involving 45 countries, affecting over 1,129,000 swine and 36,000 feral wild boars, with more than 1,931,000 animal losses (data from Immediate notifications and follow-up reports). The global outbreak of ASF has led to a sharp decline in the global swine production capacity, and the pig industry has been devastated. Due to the insidious and complex nature of ASFV infection, the current epidemic is still unclear. It is prospective that it will be difficult to recover to the previous level in 3–5 years.

**Table 1:** Number of outbreaks, cases, and animal losses caused by ASF in different regions worldwide (2020, 01–2022, 09; Source: World Organization for Animal Health, World Health Organization, October 2022 line visit).

	Outbreaks		Cases		Losses*
	Domestic swine	Wild boar	Domestic swine	Wild boar	Domestic swine
Africa	201		16,177		23,208
Americas	255		9,594		17,798
Asia	1,298	2,105	101,482	2,765	438,492
Europe	3,703	20,518	1,001,921	34,159	1,452,045
Oceania	4		500		397
Total	5,461	22,623	1,129,674	36,924	1,931,930

* Loss (number of deaths + animals killed and disposed of): this figure refers to the loss of farms affected by the outbreak and does not include animals culled in the outbreak area to control the disease.

## 3. Transmission routes of ASFV

**Figure 2:**
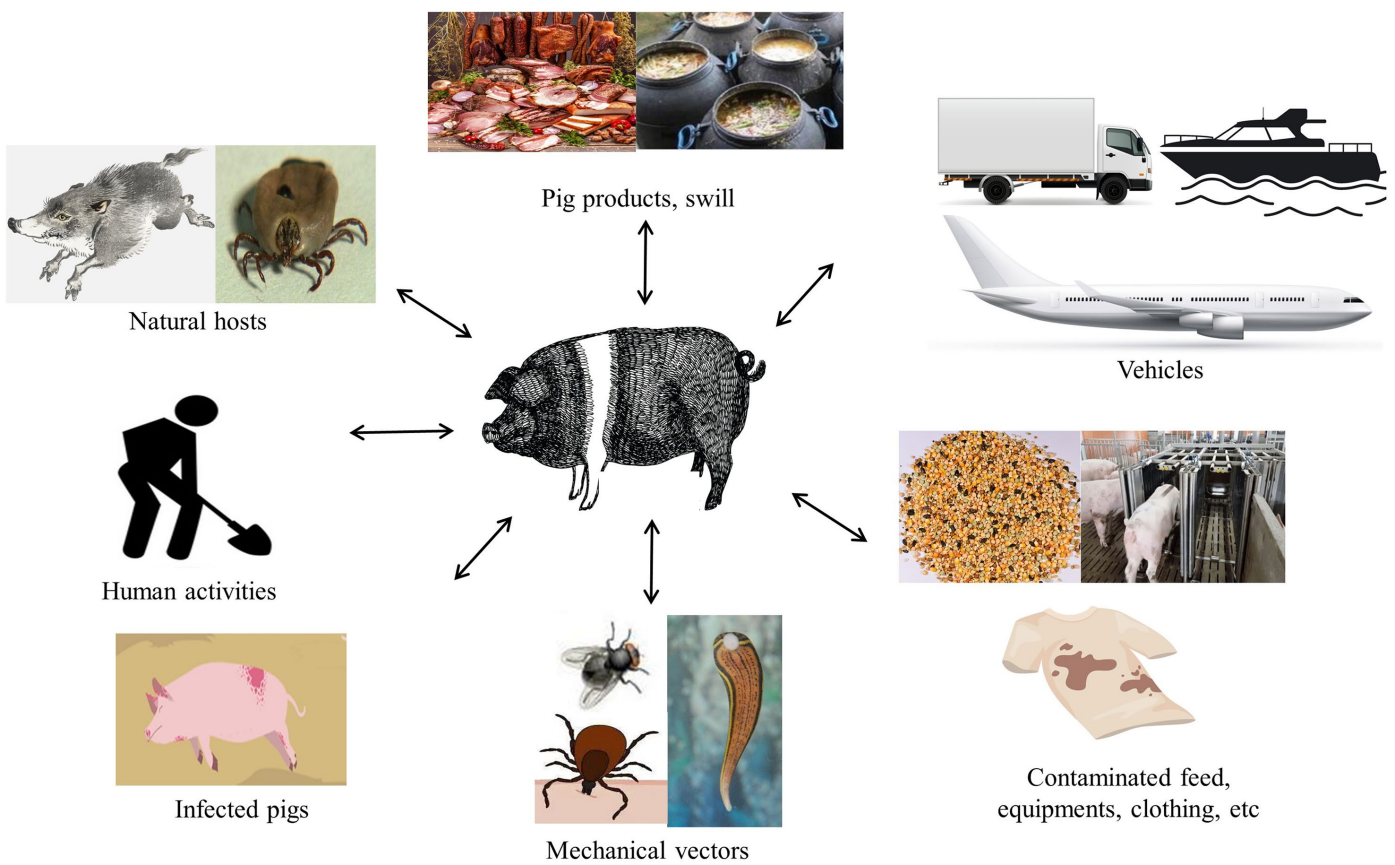
Potential ASFV routes of transmission to swine.

The ASFV of a wide range of transmission routes, including direct and indirect transmission, complicates ASF prevention and control (Figure 2). Wild boars and ticks are the natural hosts of ASFV and play a pivotal role in the spread via the forest cycle (Sánchez-Vizcaíno et al., 2015a; Śmietanka et al., 2016; Galindo and Alonso, 2017). The presence of an omnipresent herd of wild boar poses a significant barrier to the control and eradication of ASF in areas where it occurs. Sick and dead wild boars were the dominant source of the virus in Europe, which is susceptible to different ASFV genotypes, especially genotype II isolates (Blome et al., 2012), and they are vital vectors as direct or indirect carriers of the disease (Probst et al., 2017). Previous research has hinted that the virus can be transmitted by the wild-domestic swine route, which exacerbates infection in both swine and wild boars and between same-breed populations (Costard et al., 2013). In the domestic cycle, domestic swine are the sole hosts and carriers of the virus, which remains permanently infected, including swine’ feces and secretions (Guinat et al., 2016). Indeed, other animals that could also become virus carriers through mechanical transmission over long distances have been reported in several studies, such as flies, leeches, and birds, including vultures (Brown and Bevins, 2018; Olesen et al., 2018; Karalyan et al., 2019; Bonnet et al., 2020).

In addition, human activities facilitated the influx of the virus into swine farms, exacerbating the extent of virus transmission. An important route for the spread of ASFV is through the illegal transportation of infected pork products and contaminated items, including feed, equipment, vehicles, and clothing (Costard et al., 2013). Other critical factors of epidemiology, including the transport of contaminated pork products and the feeding of swilling, have caused outbreaks of ASF in the Caucasus, the Russian Federation, and China (Gogin et al., 2013). The inherent properties of ASFV are highly resistant to a wide variety of environmental conditions such as temperature, pH, and so on, resulting in the virus positively existing in the environment and sustaining to spread to distant areas (Davies et al., 2017; Schulz et al., 2017; Mazur-Panasiuk et al., 2019; Petrini et al., 2019; Zani et al., 2020). Interestingly, several studies have proven that ASFV can survive in animal feed on transatlantic routes under specific conditions (Dee et al., 2019; Stoian et al., 2019). Moreover, though frozen meat and processed pork products contaminated with the virus are not causing human infection, they may be a potential factor for virus transmission to new regions after long-distance transport due to the long-term survival of the virus (Schulz et al., 2017).

## 4. Genetic variability of ASFV

The study of the genetic evolution of the ASFV genome has entered a new phase following the sustainable development of ASFV typing techniques. Up until now, ASFV has been classified into 24 genotypes (Achenbach et al., 2017) or 8 serogroups (Malogolovkin et al., 2015). The depth of research on ASFV genotyping can facilitate tracing the origin of the virus causing the outbreak at the molecular level, understanding the potential transmission routes and possible modes of transmission, and screening potential vaccine candidates.

The ASFV genome is a linear double-stranded DNA, including left variable regions (LVR), a central conserved region (C region), and a right variable region (RVR; Van Etten, 2009). The genomic sequences of different strains may differ significantly in locations, for instance, the multigene family (MGF) within the LVR, the central variable region (CVR), and the EP402R gene (expressing the CD2v protein) within the C region. The various regions provide an advantageous condition for the evolutionary analysis of ASFV, especially for genetic evolution.

### 4.1. Genotype I strain of ASFV

Genotype I virus was first found and caused an outbreak in Portugal outside Africa in 1957 (Revilla et al., 2018). Since then it was prevalent in Portugal, Spain, Cuba, Brazil, and other countries during the 1950s to 1990s (Sánchez-Vizcaíno et al., 2015b; Galindo and Alonso, 2017). As the first case reported in the Asian region, the genotype I strain was first detected in clinical samples in China in 2021 (Sun et al., 2021). Currently, genotype I virus is still dominating in some West African countries, while no outbreak was reported in North Africa (Mulumba-Mfumu et al., 2019). In Central Africa, genotypes I and II are predominating at the same time. The specific strain information of ASFV is shown in Table 2.

**Table 2:** The specific strain information of ASFV (Source: National Center for Biotechnology Information. https://www.ncbi.nlm.nih.gov/).

Time	Country	Strain	Genbank	Gene length (bp)	Isolate gene
1957	Portugal	Lisbon 57	AF301537	415	VP72
1960	Portugal	Lisbon 60	AF301539	415	VP72
1962	Spain Espana	Madrid/62	AF449461	404	VP72
1964	France	Fr64	FJ174374	404	B646L
1968	Portugal	NH/P68	DQ028313	536	B646L
1979	Brazil	Brazil/79	AF302809	415	VP72
1979	Caribbean	DomRep/79	AF301810	392	MK4s2209
1985	Belgium	BEL/85	AF449466	404	VP72
1988	Portugal	OUR T88/3	AM712240	171,719	Complete

Portugal et al. sequenced the genomes of the high-virulence strain Lisboa60 (L60) and the low-virulence strain NH/P68 (NHV) of ASFV, comparing the differential fraction with other strains of known virulence. The analysis showed that L60 and NHV are most recently related to p72 genotype I ASFV strains from Europe and West Africa, supporting the hypothesis that the European strains originated in West Africa. Due to the long interval and geographical distance between L60 and NHV, hinting that this is due to the extensive spread of the Portuguese isolate in tissue culture (Portugal et al., 2015). The team compared gene sequences between the high-virulence Lisboa60 (L60) and the low-virulence NH/P68 (NHV) of ASFV to overcome the restrictions of virus-host interaction research, uncovering further understanding of the virus evolution (Portugal et al., 2020).

Since the introduction of genotype II ASFV in Georgia in 2007, the world has been drifting into a war against the genotype II epidemic. The ASFV genotype I strain, which differs vastly from the currently prevalent genotype II strain. Two strains of ASFV genotype I SD/DY-I/21 and HeN/ZZ-P1/21 without porcine erythrocyte adsorption activity were isolated from clinical samples of pigs in Shandong and Henan farms. Whole-genome analyses exhibited that these two strains were highly similar to the genotype I low-lethal strains NH/P68 and OURT88/3 isolated in Portugal in the 20th century and differed significantly from the genotype I high-virulent strains L60 and Benin 97 isolated in Europe and Africa in the early years. The SD/DY-I/21 and HeN/ZZ-P1/21 strains have meaningful differences in some genes despite high genome-wide sequence similarity, suggesting the possibility that they belong to different invasive sources (Sun et al., 2021). The co-existence of genotype I and genotype II strains in China emphasized that the current prevention and control situation has become more severe, accelerating the development of vaccines.

### 4.2. Genotype II strain of ASFV

The ASFV genomic study has made satisfactory progress since the rapid development of gene cloning, PCR, and sequencing technologies. p72 genotyping is the gold standard that is now widely used for ASFV genotyping. Gonzague et al. confirmed that the ASFV Malagasy strain isolated in 1999 was 99.2% related to the Mozambique strain isolated in 1994 with a highly conserved fragment of the p72 gene (Gonzague et al., 2001). In 2003, Bastos et al. first used the C-terminal end of the p72 protein to classify ASFV into 10 genotypes (Bastos et al., 2003). In 2005, Lubisi et al. further classified ASFV into 16 genotypes (Lubisi et al., 2005). In 2007, Boshoff et al. conducted a study of 43 strains of ASFV (1973–1999) isolates from South Africa (Boshoff et al., 2007). In 2016, Achenbach et al. conducted a comparative analysis of ASFV isolated in Ethiopia (2011–2014) and again identified one genotype (Achenbach et al., 2017). In 2017, Quembo et al. analyzed ASFV with 19 isolated strains from soft tick samples collected in Gorongosa National Forest Park, Mozambique. An evolutionary tree-specific analysis revealed that five strains belonged to a new evolutionary branch (Quembo et al., 2018). Thus, a total of 24 genotypes of ASFV were completely identified.

To improve our understanding of the evolutionary trends of similar strains, Gallardo et al. found that the whole ASFV genome has different combinations of tandem repeat sequences (TRS) in the intergenic region (IGR) of the I73R and I329L genes. For example, the IGR of the Polish and Lithuanian ASFV isolates was the same as that of the Belarusian and Ukrainian isolates but different from that of the Russian isolates, suggesting that the prevalent strains of ASFV in Poland and Lithuania may have originated in Belarus (Gallardo et al., 2014). Later, it was found that the virus strain in Russia had changed at the IGR site since 2012, inferring that the epidemic strain of ASFV in the EU might originate from Russia. In 2019, the virus strain (China/Guangxi/2019), which had two tandem repeat sequences at the IGR site and was primarily claimed in China, was named IGR-III (Ge et al., 2019). Subsequently, this virus strain also appeared successively in Korea (Kim et al., 2021) and Vietnam (Nguyen et al., 2022). A viral strain (Warminsko-Mazurskie, 2019) with three tandem repeat sequences, namely the IGR-IV, was also identified in Poland (Mazur-Panasiuk et al., 2020). The DNA polymerase PolX gene O174L at the 3′ end of the German ASFV pandemic strain can be distinguished in 2020 into five distinct lineages with at least 10 different mutations, suggesting that the mutation results in a pathogenic effect of the altered gene (Forth et al., 2023). The Indonesian Veterinary Science Research Center collected samples from pig farms in Bogor district, West Java province, that same year. The ASF positivity rate was 16/19, and genomic analysis revealed that this genotype of ASFV was identical to genotype II from domestic swine in Vietnam, China, and Russia (Dharmayanti et al., 2021). Senthilkumar et al. were the first to reveal the genomic analysis of the Indian strain, which is distantly linked to the Asian endemic strain. One important characteristic that could help identify the Asian endemic strain is the Mismatch between the I73R and I329L genes (Senthilkumar et al., 2022). ASFV genotyping in Sabah in 2021 revealed that this strain is comparable to the endemic strains in China, Vietnam, and Indonesia (Khoo et al., 2021).

Therefore, genomic surveillance of ASFV at the genome-wide level should be emphasized in the future, which is conducive to grasping the epidemiological pattern of ASFV, studying the disease evolution, and screening ideal vaccine candidates.

## 5. Research progress of ASFV vaccines

### 5.1. Inactivated vaccines

Inactivated ASF vaccines were first developed in the 1960s. Viral inactivation included physical and chemical methods such as heating, toluene, formaldehyde, and crystalline violet; evaluation of vaccine efficacy; and addition of adjuvants (Gómez-Puertas et al., 1997; Blome et al., 2014).

Walczak et al. indicated that anti-ASFV antibodies alone are not able to inhibit virus replication by analyzing the possibility of neutralization of the ASFV using collected sera from ASF-survivor animals (Walczak et al., 2022). However, researchers have indicated that it is impractical to use inactivated ASFV as a vaccine (Blome et al., 2014; Rock, 2017), which may be attributed to the non-neutralizing ASFV-specific antibodies (Cadenas-Fernández et al., 2021). Gomez-Puertas et al. reported that phosphatidylinositol is necessary for the correct epitope presentation of neutralizing antibodies (Gómez-Puertas et al., 1997). The viral membrane lipid composition plays an important role in antibody protein recognition, but unfortunately, it failed to detect effective specific antibodies. Multiple inactivations of inactivated vaccines do not induce effective cellular immunity in the host’s innate immune system, and with the increasing understanding of ASFV, the protective rate of inactivated ASFV vaccines is expected to improve by adding reliable adjuvants (Sang et al., 2020). The authors tested the immune efficacy of inactivated ASFV vaccines using “Polygen” and “Emulsigen D” as adjuvants, respectively, and immunized six weaned piglets twice at 21-day intervals. The animals were challenged with the homologous, extremely virulent Armenia 08 virus 42 days after the initial vaccination, both generated ASFV antibodies without neutralizing activity, and acute clinical signs appeared quickly (Blome et al., 2014). Although inactivated vaccines are antigenic, they cannot elicit a complete cellular immune response, resulting in incomplete protection (Tlaxca et al., 2015). The gamma-irradiated ASFV “Estonia 2014” has been shown to be ineffective when adjuvanted with Polygen™ or Montanide™ ISA 201 VG, respectively. Pikalo et al. demonstrated that the highly virulent “Armenia 2008” ASFV strain was not protective by inoculating weaned piglets separately with the inoculated vaccine and subjecting them to the highly virulent “Armenia 2008” strain after 42 days, showing that vaccinated animals contained specific IgG but no protection (Pikalo et al., 2022). Notably, animals inoculated with the ASFV-989 strain showed full clinical protection after 2 weeks of receiving the parental strain Georgia 2007/1. ASFV-989 could serve as a promising attenuated vaccine candidate against the challenge of homologous strains (Bourry et al., 2022). The above tests obtained similar conclusions, which may prove that currently available data suggest challenges in developing an effective inactivated ASFV vaccine using the existing methods.

### 5.2. Live attenuated vaccines

#### 5.2.1. LAVs based on naturally attenuated virus isolates

Live attenuated ASFV vaccine is developed using natural or artificial methods *in vitro*. The vaccine contains a highly viable and strongly immunogenic treated pathogen, which may increase virulence.

Some naturally weak ASFV strains can be used to develop attenuated vaccines (Chen et al., 2020). Naturally attenuated ASFV strains have been isolated from soft ticks and chronically infected swine, such as the OURT88/3, NH/P68, and Lv17/WB/Rie1 strains (Boinas et al., 2004; Gil et al., 2008; Arias et al., 2017). Live attenuated vaccines prepared from OURT88/3 were protective against homologous strains against pigs, but the protective rate was not satisfactory due to individual differences of swine, vaccination doses, and attacking strains (Chen et al., 2020). In the mid-20th century, an attenuated vaccine against ASFV serotypes I-V developed by the Federal Center for Virology and Microbiology Research protected against strains of homologous serotypes for at least 4 months on day 14 after vaccination (Sereda et al., 2020). Swine vaccinated with naturally attenuated vaccines manifest severe toxic side effects, such as fever, abortion, and chronic or persistent infection (Netherton et al., 2019). Fortunately, Gallardo et al. successfully isolated a non-HAD-ASFV (genotype II) strain, Lv17/WB/Rie1, from a wild boar in Latvia, and experimental swine, which survived received a strong hemadsorbent strain, infected with this strain showed non-specific or subclinical signs (Gallardo et al., 2019). This study reported the non-HAD-cross-protective reinfection pattern of ASFV, suggesting the long-term persistence of ASFV in wild boar herds. The fact that animals immunized with naturally attenuated strains exhibit significant side effects such as fever, skin damage, and joint swelling, which hinder the development of naturally attenuated strains of the ASFV vaccine (Leitão et al., 2001; Mulumba-Mfumu et al., 2016).

However, the immunoprotective effect of different attenuated strains varies, which is related to the type of attacking virus as well as the dose and route of administration. The naturally attenuated strain NH/P68 (genotype I) protected 100% of the virulent strain L60 (genotype I) against heterologous attack by Arm/07 (genotype II; Gallardo et al., 2018). Further, initial vaccination with OURT88/3, followed by a booster immunization with the OURT88/1 strain, resulted in 85% immune protection. Exposure to Benin 97/1 and Uganda 1965 strains resulted in 7 and 100% immune protection, respectively. Immunization against naturally attenuated strains can overcome the challenge of non-homologous ASFV strains (King et al., 2011). Subsequently, Gallardo et al. used intramuscular injection or direct contact methods to infect domestic swine with two blood-sucking ASFV (HAD strains) from Poland (Pol16/DP/OUT21) and Estonia (Est16/WB/Viru8) and three genotypes of non-HAD ASFV from Latvia (Lv17/WB/Rie1; Gallardo et al., 2021). The ASFV from Poland rapidly induced fatal and acute disease, while the ASFV from Estonia caused acute to subacute infections, with two-thirds of animals surviving. In contrast, animals infected with ASFV from virus strain of Lv17/WB/Rie1 developed a more subtle, mild, or even subclinical disease. This study provides quantitative data regarding the transmission and excretion of different strains among domestic swine and increases the focus on supervisory activities. An experiment also demonstrated that factors such as delivery route, including intramuscular and intranasal routes, and dose at low and moderate doses (10^3^ and 10^4^ TCID_50_), influence the outcome of immunization with the naturally attenuated isolate OURT88/3 (Sánchez-Cordón et al., 2017).

At present, determining whether using the Lv17/WB/Rie1 naturally attenuated strain as a vaccine prototype is an ideal strategy to control wild boar population transmission needs further research (Barasona et al., 2021). However, vaccines developed from naturally weak strains exhibit numerous side effects as well as the risk of re-dissemination of the virus, which limits their use in the clinical setting (Chen et al., 2020). Further, immune protection by natural LAVs has yet to be elucidated.

Advances in live-attenuated vaccines have recently made remarkable progress all over the world (Liu L. et al., 2021). Artificially attenuated vaccines are created by deleting specific virulence genes (O’Donnell et al., 2016; Zhang J. et al., 2021). Compared with other types of ASF vaccines, LAVs provide fully homologous and partially heterologous protection (Teklue et al., 2020a) and are ideal tools to dissect the mechanisms involved in cross-protection (Lacasta et al., 2015; Lopez et al., 2020), but virulence, immunogenicity, and, more importantly, viral phenotype and antigen diversity issues continue to affect ASF live attenuated vaccines (Revilla et al., 2018).

#### 5.2.2. LAVs based on cell passages

The cell-passaged attenuated vaccine is a LAV that causes spontaneous deletion of part of the viral genome after ASFV has been adapted to a cultured cell line, thereby reducing the virulence of the virus. In the 1960s, a laboratory study reported that a weak ASFV vaccine, weakened by transmission from porcine bone marrow (PBM) cells, protected against an attack by a strong strain. Nevertheless, Krug et al. reported that the strong strain (ASFV-G) was completely attenuated after 110 successive passages in Vero cells, and pigs immunized with the attenuated strain were not protected against it (Krug et al., 2015). In addition, ASFV passaged cultures can be adapted to cell lines such as 293 and Vero, but the virulence and antigenicity of the adapted strains are diminished, and the adapted strains do not provide effective protection after the immunization of pigs (Krug et al., 2015; Wang et al., 2021).

ASFV LAVs *in vitro* are generally based on primary cells, mainly consisting of porcine alveolar macrophages (PAMs) and PBM cells, but primary cells are not conducive to the large-scale production of LAVs. Given the failure of LAVs, additional tools are yet needed for studies involving LAVs. In 2021, Borca et al. reported that ASFV-G-ΔI177L/ΔLVR, which maintains the same attenuation level, immunogenicity profile, and protective efficacy as ASFV-G-ΔI177L, was efficiently replicated in stable porcine cell lines that could overcome the production limitations of primary porcine macrophages only (Borca et al., 2021a,b). Satisfactory discoveries revealed that the ASFV MGF-110-9L gene has a significantly reduced ability to replicate *in vitro* in primary swine macrophage cell cultures, implying the capacity of the ASFV MGF-110-9L strain for further development of ASF control strategies (Li et al., 2021a) and that a relationship involving the cGAS-STING pathway and ASFV MGF-505-7R contributed to uncovering the molecular mechanisms of ASFV (Li et al., 2021b).

Some ASFV genes’ mechanisms of action have been revealed, such as the MGF360-9L gene, which is involved in the down-regulation of interferon expression, and the I267L gene, which inhibits RNA Pol-III-RIG-I-mediated innate antiviral responses and leads to severe and lethal disease in animals vaccined (Zhang et al., 2021a; Ran et al., 2022; Zhang K. et al., 2022). Ramirez-Medina et al. assessed the role of the MGF110-5L-6L gene during virus replication in cell cultures and experimental infection in swine in 2022 and showed that deletion of MGF110-5L-6L does not impact virulence or virus replication (Ramirez-Medina et al., 2022a). In 2021 and 2022, uncharacterized protein F317L of ASFV with the function of inhibiting host innate immune response and other uncharacterized proteins EP364R and C129R with the function of inhibiting type I interferon signaling was first discovered, respectively, providing novel insights for understanding how ASFV inhibits host innate immune response and developing an ASFV live-attenuated vaccine (Yang et al., 2021; Dodantenna et al., 2022). Borca et al. claimed that the CRISPR/Cas9 gene editing system can edit target sequences in the genome with high precision, improving the purification efficiency of recombinant ASFV and providing a method for the development of recombinant ASFV LAVs (Borca et al., 2018). Although ASF LAVs have made some progress, many issues will be studied and solved in the future.

#### 5.2.3. LAVs based on deleting specific genes

##### 5.2.3.1. Single deleted genes LAVs

Approaches to the ASFV vaccine’s design currently focus on the development of modified live vaccines by targeted gene deletion from different isolates (Turlewicz-Podbielska et al., 2021).

###### 5.2.3.1.1. Benin 97/1/DP148R gene

Reis et al. found that the ΔP148R gene was transcribed early after infection and that deletion of this gene did not affect virus replication in macrophages, indicating that this gene is non-essential (Reis et al., 2017). Based on these findings, the virulent strain BeninΔP148R was obtained by deleting the gene of the virulent isolate Benin 97/1. All swine infected with the modified strain survived. Infection with BeninΔP148R caused only mild clinical signs in pigs and induced a high degree of protection against a homologous virulent virus challenge. Thus, BeninΔP148R could provide a target for rational vaccine development.

###### 5.2.3.1.2. NH/P68/A238L, A224L, A276R, EP153R

The A238L gene is involved in NFκB and NFAT regulation, the A224L gene is involved in apoptosis inhibition, the A276R gene is involved in type I interferon regulation, and the EP153R gene is a regulator of MHC-I antigen presentation. Gallardo et al. constructed recombinant NH/P68 attenuated strains by deleting these genes, respectively, with a view to developing a LAV (Gallardo et al., 2018). The results of the challenge showed that the vaccine candidate was fully effective against a homologous attack of L60 but not against genotype II Arm07, although one pig immunized with NH/P68DA224L survived.

###### 5.2.3.1.3. SY18/L7L-L11L gene

Zhang et al. constructed a deletion strain SY18∆L7-11 of the L7L-L11L gene to investigate the biology of the gene against ASFV and to gauge its potential as a vaccine candidate (Zhang J. et al., 2021). 11 of 15 pigs survived after 28 days of immunization with 10^3^ TCID_50_ and 10^6^ TCID_50_. Biological characterization indicated that deletion of the L7L-L11L gene did not affect virus replication *in vitro*. Overall, SY18∆L7-11 can be researched in more depth as a safe vaccine strain.

###### 5.2.3.1.4. ASFV-G/I177L gene

ASFV-G-∆I177L was an ideal candidate, as proved by a previous study (Borca et al., 2021a,b). Here, Tran et al. evaluated the safety of ASFV-G-∆I177L in vaccinated 6 to 8-week-old pigs, which did not cause general ASF symptoms and showed only sporadic, transient clinical signs such as mild cough and soft stools (Tran et al., 2022b). Most importantly, the virulence reduction test demonstrated the stability of the attenuated vaccine by returning five consecutive groups of pigs. Hence, ASFV-G-ΔI177L is a safe and feasible vaccine candidate. To date, the live attenuated vaccine (ASFV-G-∆I177L) developed in Vietnam is the first commercially available African swine fever vaccine in the world (Tran et al., 2022a).

###### 5.2.3.1.5. Malawi Lil-20/1, Georgia 2007, Pretoriuskop/96/4 (Pret4)/9GL gene

Back in 2000, Lewis et al. injected the retrovirus 9GL-R at 10^2^ 50% tissue culture infectious dose (TCID_50_) or mutant ∆9GL at 10^2^, 10^4^, and 10^6^ TCID_50_ into Yorkshire pigs, and when attacked with parental virulence Malawi Lil-20/1, all ∆9GL-infected animals were protected (Lewis et al., 2000). Thus, ASFV-∆9GL may prove useful as live-attenuated ASF vaccines. The ASFV Georgia 2007 strain was identified in the Caucasus and Eastern Europe by O’Donnell et al. ASFV lacking the 9GL gene was constructed and then named ASFV-G-∆9GL. Vaccinated pigs were able to resist challenges with the virulent strain after intramuscular injection of low doses of 10^2^ to 10^3^ HAD_50_ of ASFV-G-∆9GL or ASFV-G in commercial pigs (O’Donnell et al., 2015b). Additionally, deletion of the 9GL gene from the ASFV isolates Pretoriuskop/96/4 (Pret4) produced similar attenuation results (Carlson et al., 2016).

###### 5.2.3.1.6. Georgia 2010/E184L

Ramirez-Medina et al. evaluated the effect of removing the E184L gene from the ASFV-G genome on porcine virulence by injecting pigs with 10^2^ 50% of the HAD_50_ of ASFV-G-Δ184L or ASFV-G and compared them with animals infected with parentally acquired virulent ASFV-G (Ramirez-Medina et al., 2022b). The results showed that the deletion of attenuated strain E184L could distinguish between infected and inoculated animal species (DICA), but the deletion of the attenuated strain did not provide complete protection. Importantly, E184L is the first experimental ASFV gene product to function as a DIVA antigenic marker.

###### 5.2.3.1.7. BA71/CD2v gene

Monteagudo et al. reported that the knockdown of the CD2v gene in ASFV significantly reduced the virulence of BA71 strains by inoculating experimental pigs. It was not only protective against the parental strain BA71 but also the heterologous strain E75, whereas induced immune protection was dose-dependent and varied substantially among different individuals (Monteagudo et al., 2017). Bosch-Camós et al. described the immunization of pigs intranasally vaccinated with the BA71ΔCD2 deletion mutant virus, which induced an *in vitro* recall response (Bosch-Camós et al., 2022).

###### 5.2.3.1.8. Georgia/TK gene

The TK gene is associated with ASFV virulence. Based on this, Sanford et al. constructed and characterized ASFV-g/VΔTK by removing the TK gene from a Georgian ASFV strain (Sanford et al., 2016). *In vitro* experiments showed that this strain replicated in Vero cells but with a lower replication capacity than the parental strain. In addition, *in vivo* animal studies suggested that ASFVG/V-ΔTK injected with 10^6^ TCID_50_ did not cause disease in pigs but was not resistant to challenge by the parental strain.

###### 5.2.3.1.9. OURT88/3I329L

Another promising study reported the increased safety of OURT88/3 by deleting I329L, a gene that suppresses the safety of the host’s innate immune response in swine, and showed that deletion of I329L significantly reduced protection against the virulent OURT88/1 isolate (Reis et al., 2020).

###### 5.2.3.1.10. Benin 97/1/MGF

In fact, not all attenuated strains can be ideal vaccine candidates. A study concluded that naturally attenuated ASFV isolate OURT88/3 and deletion mutant BeninΔMGF were immunized in a single intramuscular dose in domestic swine, respectively, with the latter surviving longer. After 130 days of inoculation, all animals showed typical ASFV symptoms and elevated IL-10 levels. High levels of IL-10 suppressed the immune response to ASFV (Sánchez-Cordón et al., 2020). As already mentioned above, evidence suggests that regulatory components of the immune system inhibit effective protection (Franzoni et al., 2023).

Further, the protection induced by LAVs is related to the replication level of LAVs and the number of immunogenic genes expressed. If the replication of LAVs is severely impaired, the immunogenicity induced by LAVs is also diminished (Gladue et al., 2021; Zhang et al., 2021b; Tran et al., 2022a). Thus, the safety and efficacy of LAVs need to be balanced.

##### 5.2.3.2. Multiple deleted genes LAVs

Safety is the key to attenuated vaccines; therefore, vaccine developers reported a trend in the development of LAVs from single to multiple gene deletions to increase the safety of LAVs (Borca et al., 2020, 2021a,b).

Chinese scholars constructed a completely deleted 7-gene HLJ/18-7GD strain (genes encoding MGF505-1R, MGF505-2R, MGF505-3R, MGF360-12 l, MGF360-13 l, MGF360-14 l, and CD2v) using Chinese ASFV HLJ/18 as the backbone. The results of the safety and protection evaluation showed that all pigs (4/4) inoculated with HLJ/18-7GD at 10^3^ and 10^5^ TCID_50_ survived and were fully viable in the challenge with the ASFV HLJ/18 strain. Therefore, HLJ/-18-7GD can be used as a vaccine strain against ASFV (Chen et al., 2020).

The team knocked out six genes of the Georgia 2007 strain MGF360/505, containing MGF505-1R, MGF360-12L, MGF360-13L, MGF360-14L, MGF505-2R, and MGF505-3R, namely ASFV-G-MGF. *In vitro* experiments showed that the recombinant virus exhibited the same replication efficiency as the parental virus in porcine macrophages. *In vivo* experiments showed that animals injected with the recombinant virus did not develop disease and survived the challenge with the parental strain, and ASFV-G-MGF is the first experimental vaccine reported to induce protection in pigs infected with the same parental virus (O’Donnell et al., 2015a). Subsequent research suggested that deletion strains lacking the 9GL and UK virulence genes provided homologous protection only 14 days after immunization, but full attenuation was achieved through additional deletion of DP96R (UK; O’Donnell et al., 2016).

In addition, knocking out immunosuppressive genes, virulence genes, or important functional genes of ASFV using molecular biology techniques such as gene editing and reverse genetics reduces ASFV virulence and enhances the immune response (Sánchez-Cordón et al., 2018). Such as the knockdown of the MGF360/505, CD2v, and DP148R genes and so on in the ASFV genome. Further, Gallardo et al. constructed EP153R, A224L, A238L, and A276R deletion strains based on the ASFV/NH/P68 natural weak strain using a reverse genetic approach (Gallardo et al., 2018). In subsequent immunoprotection experiments, these deletion strains were found to help immunize animals against genotype I ASFV/L60 strains but failed to defend against genotype II ASFV/Arm07 strains in immunized animals (Gallardo et al., 2018); To date, the results of ASFV genetic modifications, which may be unpredictable, have been confirmed (Urbano and Ferreira, 2022). For example, the experimental vaccine strains ASFV-GΔ9GL/ΔCD2v (Gladue et al., 2020), ASFV-GΔ9GL/ΔMGF (Zhang J. et al., 2021), and ASFV-GΔ9GL/ΔNL/ΔUK (Ramirez-Medina et al., 2019), all of which had dramatically reduced protective potential compared to experimental strains lacking the individual ORFs.

Some novel recombinant viruses have been reported, including the deletion of the QP509L and QP383R genes (ASFV-ΔQP509L/QP383R) from the highly virulent ASFV CN/GS/2018 strain results in complete viral attenuation in swine (Li D. et al., 2022). The generation of a double gene-deleted ASFV mutant, ASFV-SY18-∆CD2v/UK, from a highly virulent field strain, ASFV-SY18, isolated in China, caused a loss of hemadsorption properties but did not significantly affect the *in vitro* replication of the virus in primary porcine alveolar macrophages (Teklue et al., 2020b). In the genome of the virulent ASFV isolate Benin 97/1, deletion genes of MGF360 (MGF360-10L, 11L, 12L, 13L, and 14L) and MGF530/505 (MGF530/505-1R, 2R, and 3R) and interrupting genes (MGF360-9L and MGF530/505-4R) suggested significant modulation of the IFN response on virus attenuation and induction of protective immunity (Reis et al., 2016). And the deletion of either EP402R or EP153R genes individually or in combination with BeninΔDP148R genome was shown not to reduce virus replication in macrophages *in vitro* (Petrovan et al., 2022). Furthermore, research first described that Arm/07/CBM/c4 impaired the ability to control the cGAS-STING pathway *in vitro*, similar to the NH/P68 attenuated strain, while Arm/07/CBM/c2 prevented STING and IRF3 activation (Pérez-Núñez et al., 2020).

Overall, LAVs are far from commercialized due to safety concerns. Mass vaccination can increase the risk of virulence in LAVs. Moreover, the lack of cell lines for large-scale production of LAVs is another challenge. Mutations and sequence loss in the genome of ASFV among different genotypes or even different strains of the same genotype may lead to changes in antigenicity and virulence (Bao et al., 2019). Finally, the cross-protective ability of LAVs needs to be further evaluated.

Although the development of attenuated vaccines has been unsuccessful, the Harbin Veterinary Research Institute in China has developed a seven-gene deletion attenuated vaccine strain that has completed laboratory studies, with an initial demonstration of safety and efficacy (Chen et al., 2020). Newly developed, promising LAVs candidates are summarized in Table 3.

**Table 3:** LAVs candidates.

ASFV strain	p72 genotype	Virulence	Attenuation strategy	Protection	References
Georgia 2010	II	High	Gene deleted (E184L)	Homologous strain (Georgia 2010)	Ramirez-Medina et al. (2022b)
Georgia 2010	II	High	Gene deleted (9GL, CD2v, and EP153R)	Homologous strain (Georgia 2010)	Gladue et al. (2020)
Georgia 2010	II	High	EP402R (CD2v)	Homologous strain (Georgia 2010)	Borca et al. (2020)
Georgia 2010	II	High	Gene deleted (A137R)	Heterologous strain (Georgia 2010)	Gladue et al. (2021)
Georgia 2007/1	II	High	Gene deleted (I177L)	Homologous strain (Georgia 2007/1)	Borca et al. (2021a,b)
Georgia 2007/1	II	High	Gene deleted (I177L)	Homologous strain (Georgia 2007/1)	Borca et al. (2020)
Georgia 2007/1	II	High	Gene deleted [MGF505/360(6)]	Homologous strain (Georgia 2007/1)	O’Donnell et al. (2015a)
Georgia 2007/1	II	High	Gene deleted [DP96R (UK) and B119L (9GL)]	Homologous strain (Georgia 2007/1)	O’Donnell et al. (2016)
Georgia 2007/1	II	High	Gene deleted (B119L, DP71L and DP96R)	Homologous strain (Georgia 2007/1)	Ramirez-Medina et al. (2019)
Georgia 2007/1	II	High	Gene deleted (I177L)	Heterologous strain (Georgia 2007/1)	Borca et al. (2021a,b)
Benin 97	II	High	Gene deleted (MGF505/530/360)	Homologous strain (Benin 97)	Reis et al. (2016)
Benin 97	II	High	Gene deleted (DP148R)	Homologous strain (Benin 97)	Reis et al. (2017)
SY18	II	High	Gene deleted (I226R)	Heterologous strain (Georgia 2007)	Zhang et al. (2021b)
SY18	II	High	Gene deleted (L7L-L11L)	Heterologous strain (ASFV-SY18)	Zhang J et al. (2021)
NH/P68	I	Low	Gene deleted (A276R)	Heterologous strain (virulent Arm07)	Gallardo et al. (2018)
HLJ/18	II	High	Gene deleted (MGF505-1R, MGF360-12L, MGF360-13L, MGF360-14L, MGF505-2R, MGF505-3R, and CD2v)	Homologous strain (ASFV HLJ/18)	Chen et al. (2020)
BA71	II	High	Gene deleted [EP402R(CD2v)]	Heterologous strain (Georgia 2007/1)	Monteagudo et al. (2017)
OUR T88/3	I	Low	A151R, p72, C129R, p30, p54, E146L, I215L, I73R, L8L, M448R, MGF110-4L, and MGF110-5L	Heterologous strain (OUR T88/3, OUR T88/1, Georgia 2007/1)	Netherton et al. (2019)

### 5.3. Genetically-engineered vaccines

#### 5.3.1. Subunit vaccines

ASF subunit vaccine, which primarily delivers protective antigens and antigen discovery within the ASFV genome, has recently emerged as a promising strategy against ASF (Lopera-Madrid et al., 2021). The ASFV subunit vaccine, as previously mentioned, is a potential vaccine that can effectively produce specific humoral and cellular immunity after specific gene and protein expression. Subunit vaccines are still in the laboratory phase of research, and their practical application requires further investigation. A few ASFV antigens have been shown to exhibit protective effects (Ruiz-Gonzalvo et al., 1996; Hernáez et al., 2004). For example, p30, p54, and p72 proteins are neutralizing; p72 and p54 inhibit viral adsorption; p72 and p30 activate cytotoxic T lymphocyte (CTL) responses, and p30 inhibits viral internalization (Gómez-Puertas et al., 1996). Other envelope or intramembrane proteins of ASFV, such as CD2v, p12, and D117L, may also induce neutralizing antibodies and inhibit viral invasion and release (Escribano et al., 2013).

Subunit vaccines of p30, p54, and p72 proteins were developed using a baculovirus expression system by Gómez-Puertas et al. (1996). However, after injecting the vaccines into experimental animals, effective immune protection was observed in only some of them. Subsequently, Neilan et al. showed that pigs vaccinated with a mixture of baculovirus-expressing p30, p54, p72, and p22 failed to resist the virulent strain of ASFV Pr4 (Neilan et al., 2004). In addition, recombinant baculoviruses with the ASFV hemagglutinin (HA) gene were constructed and were homologous to the T lymphocyte surface antigen CD2, and pigs immunized with recombinant HA produced hemagglutination inhibitory antibodies and temporary inhibitory antibodies, which recognized the 75-kDa structural protein and protected them from lethal infection (Gómez-Puertas et al., 1996). Based on several similarities between ASFV and HIV and poxviruses (Zhu, 2022), others have successfully prepared two p30-reactive monoclonal antibodies, 2H2 and 5E8, from mice immunized with recombinant p30 protein (Wang L. et al., 2022; Zhou et al., 2022), among the potential of genetically engineered vaccines, providing additional evidence that addresses biological challenges in subunit platform development. CD8 + T cells play an important role in protective immunity, as confirmed by the strong Portuguese ASFV strain OUR/T88/1 (Oura et al., 2005). Fan et al. also speculated that CD8 + T cell-mediated immunity plays a central role in the immune protection underlying the HLJ/18-7GD strain. The role of CD8 + T cells in ASF cellular immunity has been studied gradually (Fan et al., 2022).

In a study of immunized pigs with a fusion of genes encoding p30 and p54 proteins and the virus soluble hemagglutinin (sHA) gene, domestic swine exhibited a strong specific antibody response, which did not protect them from a strong viral attack (Argilaguet et al., 2011). However, the fusion of genes encoding the same antigens with ubiquitin resulted in the protection of 33% of immunized animals from ASFV attack (Imhof et al., 1990). The investigators also constructed an ASFV genomic expression library fused to ubiquitin (excepting for p30, p54, and the ORF for hemagglutinin), and 50 to 60% of immunized domestic swine were protected. The two pigs that survived (2/8) had no detectable virus in their blood or excreta during infection, which suggests the ability of ubiquitin to enhance class I antigen presentation and enhance CTL cell response. The protection provided by the other antigens of ASFV in the genome remains to be elucidated (Lacasta et al., 2014).

Currently, most of the ASFV protective antigens are insufficient to provide complete protection (Ivanov et al., 2011). However, Goatley et al. found that using rAd prime and MVA boosted as a delivery system with the antigens shown as immunogenic ex-ASFV, which combine eight different antigens (B602L, B646L/p72, CP204L/p30, E183L/p54, E199L, EP153R, F317L, and MGF505-5R), can protect pigs from fatal disease after challenge with a virulent genotype I strain of ASFV (Goatley et al., 2020). New strategies to develop disabled infectious single-cycle (DISC) or replication-deficient mutants as potential immunizing agents against the ASFV were proposed (Freitas et al., 2019; Urbano and Ferreira, 2022). Confirmation of the pA104R sequence demonstrates that this protein may have the potential for use in subunit vaccine design. In addition, Huang et al. screened for efficient adjuvants and provided a strategy for subunit vaccine development by constructing *Lactobacillus* p72 protein-expressing adjuvants containing IL-33 and CTA1-DD, respectively, to stimulate specific antibody production in immunized mice (Huang et al., 2022). Unfortunately, additional protective antigens need to be identified in ASFV to enable the induction of effective neutralizing antibodies to improve the immune efficacy of subunit vaccines. In our laboratory, we expressed the p30, p54, and p72 proteins encoded by ASFV *in vitro* using the *Lactobacillus lactis* expression system. Findings suggested that recombinant *Lactobacillus* induced humoral, cellular, and local mucosal immunity via orally administered rabbits at a dose of 10^8^ CFU/ml of combined immunization (Zhang et al., 2023).

Notably, Mazloum et al. accelerated the understanding of the effect of proteins with unknown functions on ASF virus replication in the CV-1 cell line by evaluating recombinant plasmids pCI-neo/E248R, pCI-neo/EP402R, and pCI-neo/X69R (Mazloum et al., 2019). Thus, Several recombinant MVA vectors were constructed by Lopera-Madrid et al. (2021) to evaluate the efficiency of different promoters and secretion signal sequences on ASFV p30 protein expression and immunogenicity. These results indicate that promoter selection may be as vital as the antigen used to develop ASFV subunit vaccines. The satisfactory results of which were evaluated for humoral and cellular immunity in mice by two recombinant fusion proteins, OPM (OprI-p30-modified p54) and OPMT (OprI-p30-modified p54-T cell epitope), suggested that OPMT might be an ideal candidate to elicit immune responses in swine (Zhang G. et al., 2022).

#### 5.3.2. DNA vaccines

Although DNA vaccines can induce high levels of specific T-cell responses in the host (Argilaguet et al., 2012; Bosch-Camós et al., 2020), immunized animals are still not fully resistant to strong strain attacks. Argilaguet et al. confirmed the poor immunogenicity of DNA vaccines in large animals (Argilaguet et al., 2011). The immunized pigs with plasmid DNA encoding two ASFV gene frames (pCMV-PQ), failed to elicit an immune response in pigs. They were, however, successful in mice. Subsequently, a new recombinant plasmid, pCMV-APCH1PQ, was constructed to improve immunization in pigs. The results hinted that the DNA vaccine did not protect against lethal viral attack, but targeting the antigen to antigen-specific cells significantly enhanced the immune response in pigs. A similar study used a vaccine prepared from an ASFV genomic expression library to immunize breeding pigs, which provided 60% protection against attack by the highly virulent E75 strain (Ivanov et al., 2011). An important strategy investigated the ability of class I molecular antigen presentation and enhancement of the immune response after CTL induction and the construction of a recombinant plasmid PCMV-UbsPQ, confirming the potential of T-cell responses in the prevention of ASF and the development of effective recombinant vaccines in the future (Argilaguet et al., 2012).

The ASF DNA vaccine made using p54/p30 as the target antigen does not produce neutralizing antibodies in pigs or generate cellular immunity (Jancovich et al., 2018). There are some unsatisfactory results, such as the DNA vaccine developed by p54/p30/sHA fusion (Chen et al., 2021), plasmid DNA (CD2v + p72 + p32 and +/−p17), and recombinant proteins (p15 + p35 + p54 and +/−p17) in a cocktail immunization strategy (Sunwoo et al., 2019), the co-immunization using recombinant protein pCD2-E and recombinant plasmid pcDNA312R (Pérez-Núñez et al., 2019; Sunwoo et al., 2019), and a gene fragment encoding the extracellular region HA of ASFV using p54 and p30, all results of above mentioned had no protective capacity against ASFV of pigs (Chen et al., 2021). While the DNA-protein vaccination strategy tested was not effective, it also triggered an earlier onset of clinical signs, viremia, and death after a virulent ASFV challenge compared to non-immunized pigs (Sunwoo et al., 2019). However, a strong CD8 + T cell response was induced by co-immunizing pigs with the ubiquitin gene and the p30 and p54 genes, providing partial protection even in the absence of specific antibodies, suggesting that DNA vaccines enhance cellular immune responses by modifying the antigens (Jancovich et al., 2018; Goatley et al., 2020).

Interestingly, the authors used the concordant sequences of ASFV antigens p12, p17, p22, p54, p72 and CD2v genotypes to predict B-cell, helper T-cell, and cytotoxic T-cell epitopes and conjugated them with adjuvants and linkers to form ASF vaccines (Buan et al., 2022). Immunostimulation experiments showed that the designed ASF vaccine stimulated immune cell effects and cytokine production. Here, Bosch-Camós et al. used data from the SLAI-peptide repertoire presented by a single set of ASFV-infected porcine alveolar macrophages to create a complex DNA vaccine against the Georgia2007/1 lethal challenge, composed of 15 plasmids encoding the individual peptide-bearing ORFs (Bosch-Camós et al., 2021). The result confirmed that two proteins, DNA plasmids encoding M488R and MGF505-7R, a CD8 + T-cell antigen previously described, have T-cell antigens with protective potential. Freitas et Al. described a feasible approach to generating safe and efficient DISC vaccine candidates of ASFV by homologous recombination, in which the A104R gene was replaced by a selection marker (GUS gene; Freitas et al., 2019). In brief, the results suggest that the designed multi-epitope and multi-antigen ASF vaccine requires further exploration.

#### 5.3.3. Vector vaccines

Viral vectors are an emerging type of vaccine in which the internal genome of the viral particle is genetically engineered to carry one or more antigenic genes of the target virus, which can inhibit virus replication. A review described that proteins of p30, pp62, p54, p72, and CD2v were designed as approachable viral vectors because of their strong immunogenicity and the ability to induce the body to produce neutralizing antibodies in the course of the virus cycle (Ravilov et al., 2022). Furthermore, viral vectors can distinguish between infected and vaccinated animals, i.e., the immunogen encoded by the viral vector can be used as a vaccine marker (Gaudreault and Richt, 2019).

Significant discoveries in recent years addressed partly the limitations of traditional inactivated and attenuated virus vaccines. Some authors have based their studies on vectors for gene transfer into mammalian cells, such as by constructing a baculovirus-based gene transfer vector, BacMam-sHAPQ, which is a potential tool for future vaccine development (Argilaguet et al., 2013). Another promising study evaluated two different adjuvants and two immunometric formulations of adenoviral vector (Ad-ASFV) multi-antigens for safety and immunogenicity, which demonstrated for the first time that the Ad-ASFV multi-antigens can be used to induce ASFV anti-specific CTL responses (Lokhandwala et al., 2016). Subsequently, the team also evaluated the protective effect of the adjuvant by intranasal injection of ASFV-Georgia 2007/1 in pigs based on a previous study, which did not result in significant protection (Lokhandwala et al., 2019). Moreover, the immunogenicity of seven adenoviral vectors of ASFV neoantigens (A104R, A151R, B602L, B438L, B119L, EP402RΔPRR, and K205R) was evaluated. Another strategy followed to induce local and systemic immunity via a cocktail of recombinant adenoviruses formulated with adjuvants in pigs was also described (Lokhandwala et al., 2017).

Currently, the antigens for primary and booster immunization are mostly used in subunit vaccines, DNA vaccines, and viral vector vaccines via cross-immunization strategies. For example, p72, p30, p54, E183L, E199L, EP153R, F317, and MGF505-5R recombinant viruses were constructed using poxvirus and adenovirus vectors. Pigs immunized and exposed to Benin97/1 using recombinant poxvirus for primary immunization and recombinant adenovirus for booster immunization represent the best combination of viral vector antigens for immunization to date (Freitas et al., 2019). Chen et al. assessed the immunoassay of piglets and mice using the recombinant virus rBartha-K61-pASFV (Chen et al., 2022). Murgia et al. used an alphavirus vector platform to deliver replicon particles (RPs) expressing ASFV antigens to swine (Murgia et al., 2019). Feng et al. evaluated the effectiveness and safety of recombinant pseudorabies virus [PRV; PRV-ΔgE/ΔgI/ΔTK-(CD2v)] in mice (Feng et al., 2020). Fang et al. established a Semliki Forest virus (SFV) vector expressing ASFV p32 (SFV-p32) and p54 (SFV-p54; Fang et al., 2022). These results highlighted the possibility of developing ASF vaccines using a rational viral vector.

The CRISPR/Cas9-based gene editing technology proposed by Hübner et al. (2018) provides a new direction for screening potential immunoprotective antigens and for developing effective ASFV vector vaccines. Chen et al. constructed and expressed recombinant ASFV p72 protein by reverse genetics using the Newcastle disease virus and evaluated its humoral and cellular immunogenicity in a mouse model but the protective effect of this vaccine in pigs needs further study (Chen et al., 2016). Interestingly, immunization of wild boars with various antigens (p32, p54, p72, and pp62) expressed in adenovirus induced strong IgGs, IFN-γ, and CTL responses but did not protect against intranasal attack by the ASFV-Georgia 2007/1 strain (Cadenas-Fernández et al., 2020). A strategy incorporating priming with a vector-expressed antigen followed by boosting with an attenuated live virus may broaden the recognition of ASFV epitopes (Murgia et al., 2019). The protective effect of the vector vaccine constructed by Lopera-Madrid et al. remains to be validated. Results of above mentioned showed that limited success in this field attained in previous studies, and protection of vaccines for pigs remains a focus of future research (Lopera-Madrid et al., 2017).

In the future, the design of recombinant vector vaccines will use emerging biological technologies such as gene recombination, reverse genetics, and CRISPR/Cas9 gene editing. Meanwhile, the identification of ASFV virulence genes, functional genes for viral replication, and key genes regulating the host immune response should be encouraged. We expect to combine different viral vectors and multiple efficient protective antigens or genes to construct safe and effective recombinant vector vaccines or DNA vaccines. Newly developed, promising genetically engineered vaccines are summarized in Tables 4, 5.

**Table 4:** ASF subunit and DNA vaccines.

Vaccine type	Sequence source	Gene/protein	Specific antibodies	Neutralizing antibodies	References
Subunit vaccines	E75CV	HA (CD2v)	Yes	No	Ruiz-Gonzalvo et al. (1996)
	Krasnodar 07/17 ASF/ARRIAH/CV-1	CD2v, pE248R and pX69R	Yes	Yes	Mazloum et al. (2019)
	SY18	p30 and p54	Yes	Yes	Zhang G. et al. (2022)
	Pr4	p54, p30, p72 and p22	Yes	Yes	Neilan et al. (2004)
	E70	Group1: p158, p327, p14 and p220; Group3: p30 and p72	No	/	Ivanov et al. (2011)
	Georgia 2007/1	p30	Yes	No	Lopera-Madrid et al. (2021)
		ASFV-pA104R	/	/	Freitas et al. (2019)
		p14.5, p14.5-IL-33-Mus f and CTA1-p14.5-D-D	Yes	/	Huang et al. (2022)
DNA vaccines	E75	SLA-II/p54/p30 fusion	Yes	No	Argilaguet et al. (2011)
		sHA/p54/p30 fusion	Yes	No	Argilaguet et al. (2012)
		Ub/sHA/p54/p30 fusion	/	/	Argilaguet et al. (2012)
		p54, p30, and the hemagglutinin extracellular domain/sHA	No	/	Ivanov et al. (2011)
	Georgia2007/1	M448R and MGF505-7R	Yes	Yes	Bosch-Camós et al. (2021)
		p30-Fcc and p54-Fca	Yes	/	Chen et al. (2021)
		47 antigens	Yes	No	Jancovich et al. (2018)
	Ba71V	80 ORFs fragments fused with Ub	Yes	NA	Lacasta et al. (2014)
	Armenia 2007	DNA: CD2v, p72, p30, +/−p17; Proteins: p15, p35, p54, +/−p17	Yes	No	Sunwoo et al. (2019)
	OUR T88/3Benin 97/1	p72, p30, p54, E183L, E199L, EP153R, F317L and MGF505-5R	Yes	Yes	Goatley et al. (2020)
	E70; Ba71V	DNA:CD2v, p30, p72, CP312R; Proteins: p15, p35, p54, p72 and CD2v-E (sHA)	Yes	Yes	Pérez-Núñez et al. (2019)
	NCBI virus database	p12, p17, p22, p54, p72 and CD2v	/	/	Buan et al. (2022)

**Table 5:** ASF vector vaccines.

Vaccine type	Sequence source	Gene/protein	Specific antibodies	Neutralizing antibodies	References
Vector vaccines	E75	sHA/p54/p30 fusion	No	No	Argilaguet et al. (2013)
	Georgia 2007/1	p30, p54, pp62, and p72	Yes	No	Lokhandwala et al. (2016)
		A151R + B119L + B602L + EP402R∆PRR + B438L + K205R + A104R	Yes	No	Lokhandwala et al. (2017)
		Ad-ASFV-I: A151R, B119L, B602L, EP402R∆PRR, B438L, K205R, A104R, pp62 and p72 Ad-ASFV-II: p30, p54, pp62, p72 and pp220 (p37-34-14, p150-I and p150-II)	Yes	/	Lokhandwala et al. (2017)
		pE199L	/	/	Hübner et al. (2018)
		p72	Yes	/	Chen et al. (2016)
		35 antigens	Yes	/	Cadenas-Fernández et al. (2020)
		p72, p54, p12 and p72, type Lectin (EP153R), CD2v and p72, C-type Lectin (EP153R) and CD2v	Yes No /	No / /	Lopera-Madrid et al. (2017)
	NCBI Virus Database	p30, pp62, p54, p72 and CD2v	Yes	/	Chen et al. (2022)
		p30, p54 and p72	Yes	Yes	Murgia et al. (2019)
		p32 and p54	Yes	/	Fang et al. (2022)
	HLJ/2018	CD2v	Yes	/	Feng et al. (2020)

## 6. Conclusion

Since January 2020, ASF has been reported in 5 different regions involving 45 countries, resulting in more than 1,931,000 animal losses. The global outbreak of ASF has led to a sharp decline in the global swine production capacity, and the pig industry has been devastated. Due to the insidious and complex nature of ASFV infection, the current epidemic is still unclear. In the long run, coordinating the development of all countries and regions worldwide is critical to relieving the pressure from the ASF epidemic. To commercialize a satisfactory ASF vaccine, however, a variety of challenges must be addressed as a matter of priority. Regarding the authors’ published studies, several current problems and future directions of ASF vaccines were summarized as follows:

### 6.1. Current problems of ASF vaccines

The genome of ASFV is very large, ranging from 170 to 193 kb and encoding 150 to 167 genes with virulence and immune-related functions which is one of the reasons why ASF vaccines are difficult to commerce. Previously, Burmakina et al. proved that ASFV serotype-specific proteins CD2v (EP402R) and/or c-type lectin (EP153R) are important for protection against homologous ASF infection (Burmakina et al., 2019), but the complexities of synergistic interactions among multiple genes are poorly understood (Rowlands et al., 2009; Sánchez-Vizcaíno et al., 2015b; Dixon et al., 2019; Simões et al., 2019). According to the VP72 protein gene B646L, the strains that have lately been common in China, Southeast Asia, and Europe include natural mutations or disappearance mutations, which increase the uncertainty of genome stability (Faburay, 2022). Prior studies have noted the importance of ASFV growing stably and rapidly *in vitro*. Thus, in-depth research on ASF genomics and the function of different genes, including those related to the immune response, is vital to developing an ASF vaccine.

Due of the quick demise of sensitive animals when exposed to virulent strains, previous investigations have been unable to establish a connection between viruses and hosts. Most of the experiments have confirmed that inactivated vaccines led to further expansion of the epidemic and live attenuated vaccines caused a persistent infection, detoxification, and side effects in immunized pigs, while there was still controversy over whether ASFV infection produced neutralizing antibodies. In addition, the low cross-protection between various strains makes screening vaccine candidates challenging (Muñoz-Pérez et al., 2021). According to Qu and colleagues’ explanation, this may help pinpoint the virus’s origin in an outbreak and may aid in the development of an ASF vaccine (Qu et al., 2022). Up to this point, pigs and biosafety level-3 (BSL-3) laboratories restrict the vaccine’s wide development that why it is difficult to obtain reliable animal models for evaluating the immunological effects of vaccines. A numuber of studies sum up the procedures used to ensure the safety of the vaccination and assess the viability of using animals in experiments (Sánchez et al., 2019; Dixon et al., 2020; Sang et al., 2020; Wu et al., 2020). Unfortunately, recent instances of attenuated strains on Chinese farms have led to chronic illnesses, clinical side effects in pig herds, and industrial threats (FAO, 2021). Live virulence vaccines’ safety is questioned due to unregistered, illegally attenuated strains.

### 6.2. Future directions of ASF vaccines

Biosecurity may be the most crucial method to resist the spread of ASF until an effective vaccine is developed. Based on underlying ASF risk factors, it was possible to group the present pandemic’s risk factors into the following categories: ASFV, biosecurity, disease control, environment, livestock, sports, network, pig, society, and surveillance (Bergmann et al., 2021). Advancing studies on the potential contribution of mechanical vectors, such as human activity, arthropods, birds, and carnivores, to ASF transmission, are critical (Wu et al., 2020). Besides, it may be worthwhile to investigate the involvement of environmental factors in the development of ASFV. Recently, a successful disease dynamics model was described to explain how ASFV spreads in Vietnamese pig herds and to prevent outbreaks in the future (Mai et al., 2022). Penrith et al. analyzed certain porcine-associated viruses of genotype II, which tended to widen their geographic distribution; meanwhile, the role of ASF survivors as virus carriers, as well as the duration of immunity, still has to be further investigated (Penrith and Kivaria, 2022).

Now, designing some promising methods for distinguishing between infected and vaccinated animals using the distinction between infected and vaccinated animals (DIVA) strategy is necessary for ASFV vaccination campaigns. One of those is a three-independent real-time polymerase chain reaction (qPCR) method to support vaccination campaigns associated with ASFV-ΔMGF, ASFV-G-Δ9GL/ΔUK, and ASFV-ΔI177L or ASFV-ΔI177LΔLVR cell culture live attenuated vaccines (Velazquez-Salinas et al., 2021), and the other is a double indirect ELISA based on p54 and CD2v (Wang Z. et al., 2022). Beyond that, the ASFV E248R gene was selected to be the target for establishing a real-time PCR method, which is used for the efficient detection of infected ASFV and PRRSV live vector viruses expressing ASFV antigen protein (Li L. et al., 2022). Wu et al. (2022) developed a real-time recombinase-aid amplification (RAA) assay to rapidly detect the different genotypes of ASFV, including the E70 strain (Spanish), the Anhui XCGQ strain, and the Georgia 2007/1 strain and contributed to the development of a control strategy for ASF (Wu et al., 2022). These details could additionally shed light on potential protocols to prevent and control ASF.

Here, we reviewed the preliminary evidence that a safe and effective attenuated vaccine strain with seven gene deletions and Lv17/WB/Rie1 protection against challenge with a virulent ASF virus isolate exists (Barasona et al., 2019). To date, the antigenic and protective properties of two attenuated ASFV strains, MK200 and FK-32/135 (Sereda et al., 2022), evaluated pig samples immunized with a single dose of 10^6^ TCID_50_ HLJ/18-7GD (Fan et al., 2022), described the immunization of pigs intranasally inoculated with a BA71ΔCD2 deletion mutant virus, induced *in vitro* recall responses (Bosch-Camós et al., 2022), and found that ASFV-ΔA137R induced higher production of type I interferon (IFN) in PAMs (Sun et al., 2022). Moreover, the design of vaccines intended for wild boars and oral administration is desirable, although these vaccine candidates are still in the preliminary phase and further field experiments need to be carried out. As mentioned earlier, in addition to using numerous ASFV antigens or antigen fragments and optimizing the choice of adjuvants and chemical formulations, attention should be paid to the protective antigens of existing strains and their ideal immunological mechanisms.

A vaccine is, and always will be, the essential strategy, and the best vehicle for preventing and controlling anti-ASFV. Future research on ASFV’s virology and functional genomics will focus on topics like protein structure and function, mechanisms of infection and immunity, identification of additional protective antigens as immunogens, targets for vaccines to boost immune protection, and thorough testing in target animals. Safety and immune effect also should be evaluated in future studies.

## Author contributions

HoZ and SZ wrote the first draft of the manuscript and looked up the relevant literature. XC and HS guided manuscript writing. HaZ and ZQ performed writing-review and editing. All authors contributed to the article and approved the submitted version.

## Funding

This work is based upon research funded by the Shandong Provincial Key Research and Development Program (Major Scientific and Technological Innovation Project; No. 2020CXGC010801-02), the Shandong Province Agricultural Major Application Technology Innovation Project (No. SD2019XM003), the Project of Shandong Province Science and Technology Achievement Transfer Transformation Subsidy (No. 2021 LYXZ020), the Shandong Provincial Major Project of the New-Old Kinetic Energy Conversion [No. (2020)1220], and the Project ZR2020MC185 supported by Shandong Provincial Natural Science Foundation.

## Conflict of interest

HZ was employed by the company Qingdao Haihua Biological Group Co., Ltd, Qingdao, China.

The remaining authors declare that the research was conducted in the absence of any commercial or financial relationships that could be construed as a potential conflict of interest.

## Publisher’s note

All claims expressed in this article are solely those of the authors and do not necessarily represent those of their affiliated organizations, or those of the publisher, the editors and the reviewers. Any product that may be evaluated in this article, or claim that may be made by its manufacturer, is not guaranteed or endorsed by the publisher.
