# Corrigendum: Vaccines for African swine fever: an update

**DOI:** 10.3389/fmicb.2024.1529175

**Published:** 2024-12-06

**Authors:** Hongliang Zhang, Saisai Zhao, Haojie Zhang, Zhihua Qin, Hu Shan, Xiulei Cai

**Affiliations:** ^1^Shandong Collaborative Innovation Center for Development of Veterinary Pharmaceuticals, College of Veterinary Medicine, Qingdao Agricultural University, Qingdao, China; ^2^College of Animal Science and Technology, Shandong Agricultural University, Tai'an, China

**Keywords:** African swine fever virus, epidemic and spread, vaccine, progress, review

In the published article, there was an error in the Funding statement. The last funding number is incorrect. The correct Funding statement appears below.

